# Properties and Mechanism of PEALD-In_2_O_3_ Thin Films Prepared by Different Precursor Reaction Energy

**DOI:** 10.3390/nano11040978

**Published:** 2021-04-10

**Authors:** Ming-Jie Zhao, Zhi-Xuan Zhang, Chia-Hsun Hsu, Xiao-Ying Zhang, Wan-Yu Wu, Shui-Yang Lien, Wen-Zhang Zhu

**Affiliations:** 1 School of Opto-Electronic and Communication Engineering, Xiamen University of Technology, Xiamen 361024, China; 2015000077@xmut.edu.cn (M.-J.Z.); 1922031023@stu.xmut.edu.cn (Z.-X.Z.); chhsu@xmut.edu.cn (C.-H.H.); xyzhang@xmut.edu.cn (X.-Y.Z.); wzzhu@xmut.edu.cn (W.-Z.Z.); 2 Fujian Key Laboratory of Optoelectronic Technology and Devices, Xiamen University of Technology, Xiamen 361024, China; 3 Department of Materials Science and Engineering, Da-Yeh University, Changhua 51591, Taiwan; wywu@mail.dyu.edu.tw

**Keywords:** indium oxide, plasma-enhanced atomic layer deposition (PEALD), substrate temperature, high growth rate, precursor reaction energy

## Abstract

Indium oxide (In_2_O_3_) film has excellent optical and electrical properties, which makes it useful for a multitude of applications. The preparation of In_2_O_3_ film via atomic layer deposition (ALD) method remains an issue as most of the available In-precursors are inactive and thermally unstable. In this work, In_2_O_3_ film was prepared by ALD using a remote O_2_ plasma as oxidant, which provides highly reactive oxygen radicals, and hence significantly enhancing the film growth. The substrate temperature that determines the adsorption state on the substrate and reaction energy of the precursor was investigated. At low substrate temperature (100–150 °C), the ratio of chemically adsorbed precursors is low, leading to a low growth rate and amorphous structure of the films. An amorphous-to-crystalline transition was observed at 150–200 °C. An ALD window with self-limiting reaction and a reasonable film growth rate was observed in the intermediate temperature range of 225–275 °C. At high substrate temperature (300–350 °C), the film growth rate further increases due to the decomposition of the precursors. The resulting film exhibits a rough surface which consists of coarse grains and obvious grain boundaries. The growth mode and properties of the In_2_O_3_ films prepared by plasma-enhanced ALD can be efficiently tuned by varying the substrate temperature.

## 1. Introduction

Indium oxide (In_2_O_3_) thin film combines excellent optical properties with excellent electrical properties, which makes it suitable for application in a multitude of optoelectronic devices. For instance, tin doped In_2_O_3_ (ITO) film is a typical transparent conductive oxide (TCO) that can be used as the transparent electrodes of many optoelectronic devices such as liquid crystal displays (LCDs), organic light-emitting diodes (OLEDs), and solar cells [1,2,3]; In_2_O_3_-based semi-conductive films represented by indium zinc oxide (IZO), and indium gallium zinc oxide (IGZO) have been utilized as the channel material of thin film transistors (TFTs) for active-matrix flat panel displays [4,5].

Many methods have been used to prepare In_2_O_3_ thin film, including sputtering [6], chemical vapor deposition (CVD) [7], sol–gel process [8], and atomic layer deposition (ALD) [9,10,11,12,13]. Among these methods, ALD is specialized in producing thin film with exceptional conformality and uniformity owing to the self-limited surface reaction that allows the precise control of the film growth to the atomic-layer-scale level. Many efforts have been made to prepare In_2_O_3_ film through the ALD method. However, the film growth rate was usually low (typically 0.065–0.83 Å/cycle) when conventional oxidants such as water (H_2_O) and oxygen gas (O_2_) were used due to the low reactivity of most of the available indium precursors to these oxidants [9,10,12]. In addition, a narrow ALD process window was also observed for some ALD-In_2_O_3_ processes due to the poor thermal stability of the indium precursors [9,14]. For instance, the ALD windows were 200–251 °C, 160–200 °C when using precursor pairs of TMIn and H_2_O, InCp and H_2_O_2_, respectively [9,14]. Developing indium precursors with high reactivity and thermal stability is an important strategy for achieving a reasonable film growth rate and extending the ALD process window [15]. In addition, utilizing stronger oxidants, such as ozone (O_3_) and O_2_ plasma is a feasible approach to enhance the reaction of precursors, hence significantly increasing the growth rate of ALD-In_2_O_3_ film [13,16]. Recently, In_2_O_3_ film has been successfully prepared by ALD with the assistance of O_2_ plasma [16,17,18]. In these studies, the influence of deposition temperature in the range of 70–400 °C on the In_2_O_3_ film properties and the performance of the TFT device have been investigated, in the aim of capturing the ALD window and achieving suitable or optimal characteristics for device application. Therefore, more attentions have been paid on the optimization of the film properties. The optimal deposition temperature depends on what precursors were used. For instance, the optimal deposition temperature ranges are 200–250 °C, 100–400 °C (wide ALD window but very low deposition rate of 0.14 Å/cycle) and 90–180 °C for Et_2_InN(SiMe_3_)_2_, In(TMHD)_3_ and Me_2_In(EDPA) precursor, respectively. However, the film growth mechanism and its relation to the film properties are rarely discussed, especially beyond the ALD window. In fact, the deposition temperature is crucial for determining the film growth mode as it would affect the adsorption state on the substrate and the reaction energy of the indium precursors, and even cause the thermal decomposition of the precursors at high temperature. Therefore, the film growth mechanisms should be dependent on the deposition temperature. However, the relations between the film growth mechanisms and the film properties at a different deposition temperature range have not been systematically investigated and well established. In this work, In_2_O_3_ films were prepared by an O_2_ plasma-enhanced ALD (PEALD) process. The film growth mechanisms of the PEALD-In_2_O_3_ thin films in a wide deposition temperature range were discussed. The relations between the film growth mechanisms and the film characteristics were systematically investigated. This would be beneficial for a deeper understanding of the film growth and the tuning of the film properties by deposition temperature.

## 2. Materials and Methods

**Figure 1:**
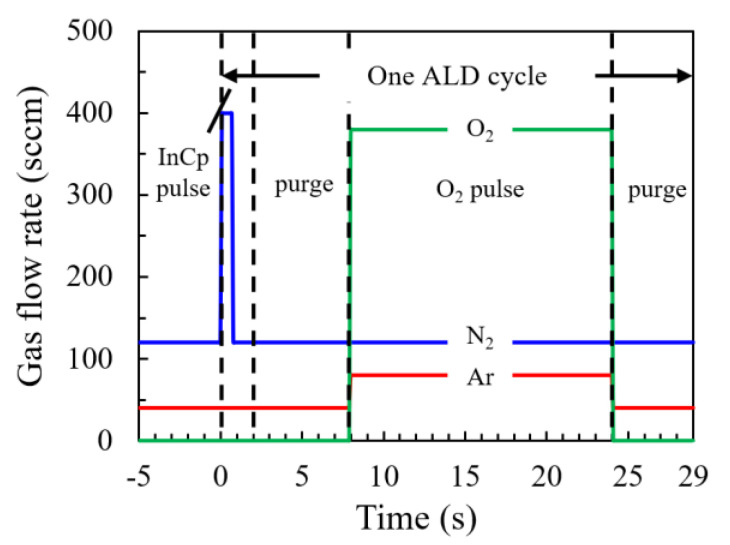
The flow rates of gases in the atomic layer deposition (ALD) process with respect to the time sequence.

In_2_O_3_ films were prepared by a PEALD system (R-200, Picosun, Espoo, Finland) using cyclopentadienyl indium (InCp, Aimou Yuan, Nanjing, China) as the indium precursor. The indium precursor bubbler was maintained at a temperature of 90 °C which is higher than the sublimation temperature of InCp (40 °C) to provide sufficient vapor pressure [13,14]. Nitrogen (N_2_) gas was used as both the carry gas (120 sccm) and dilute gas (400 sccm) of the indium precursors. The flow rates of the gases with respect to the time sequence were plotted in Figure 1. Before starting the ALD process, N_2_ gas in a total flow rate of 120 sccm was injected into the reaction chamber from six spray heads distributed in the upper corner of the chamber and Argon (Ar) gas in a flow rate of 40 sccm was injected from a spray head on the right top of the chamber. Meanwhile, the gas was pumped out of the chamber from the bottom by a pump system. In this way, the chamber was kept clean by filing of inert gas. A base pressure of about 100 Pa was achieved after the balance of the gas injection and pumping out before starting the ALD process and kept throughout the whole process with negligible influence from the precursor pulses. An ALD cycle can be described as follows: firstly, the indium precursor was diluted by N_2_ gas of 400 sccm for 0.7 s, then the diluted indium precursor was carried into the chamber by N_2_ gas (120 sccm) for 1.3 s and adsorbed on the substrate; then, a purge step was executed with a pulse time of 6 s using N_2_ (120 sccm) and Ar (40 sccm) gas; next, a mixture gas of Ar and O_2_ with a respective flow rate of 80 and 380 sccm was injected into the chamber and discharged by an inductive coupled RF power of 3000 W to generate O_2_ plasma working in a remote mode with a distance of about 60 cm between the coil and the substrate surface; finally, a purge step was executed with a pulse time of 5 s. The pulse durations mentioned above have been tuned aiming at reaching a saturated film growth rate or removing the redundant precursor and reaction byproduct. The films were grown by repeating the ALD cycle as described above. Four hundred cycles were executed in total for each specimen. The substrate temperature was varied from 100–350 °C to investigate its influences on the mechanism of the film growth and the film properties. The parameters for the PEALD process were summarized in Table 1.

In_2_O_3_ films were grown on silicon wafer for the evaluation of the thickness (*t*), refractive index (*n*) and extinction coefficient (*k*) of the film by a spectroscopic ellipsometer (M-2000, J. A. Woollam Co., Inc., Lincoln, NE, USA). An air roughness model consisting of a four-layer structure of “air, air/In_2_O_3_, In_2_O_3_ and silicon wafer”, where the In_2_O_3_ layer was fitted by a Tauc–Lorentz model was used to fit the data. The light absorption coefficient (α) was calculated from the extinction coefficient using the following equation [19]:

α = 4πk/*λ* (1)

where is λ the wavelength of the incident light. The optical band gap (*E*_g_) of the In_2_O_3_ films was deduced by fitting the Tauc plot [20] as expressed by the equation:

(αh*ν*)^2^ = A(h*ν* − *E*_g_) (2)

where α is the light absorption coefficient of the In_2_O_3_ film, h*ν* is the energy of the incident light, A is a constant. The crystal structure of the films was investigated by grazing incident X-ray diffraction (GIXRD, Rigaku TTRAX III, Ibaraki, Japan) spectra with an X-ray wavelength of 0.154 nm and an incident angle of 1°. The average sizes (*D*) of the (2 2 2)-orientated crystallites were calculated from the full width at half maximum (FWHM, *β*) of the (2 2 2) peak according to Scherrer’s equation [21]. The FWHM was extracted using an analysis software (MDI Jade 6) with the instrumental broadening deducted automatically. The morphology of the film surface was observed by field emission scanning electron microscopy (FESEM, Sigma 500, Zeiss, Germany) and atomic force microscopy (AFM, Bruker, Billerica, Massachusetts, USA). The operation voltage for the SEM observation is 15 kV. The AFM was working in the taping mode with a scan rate of 0.75 Hz during the measurement. The constituents and chemical states of oxygen element in the film were analyzed by X-ray photoelectron spectroscopy (XPS, ESCALAB 250Xi, Thermo Fisher, Waltham, MA, USA). The film surface was pre-sputtered by an Ar ion beam spot of 2 mm×2mm for 0.5 min before recording of the XPS spectra to exclude the influence of surface contamination. The accelerating voltage for the ion beam was set to be 2 kV. The sputter rate was estimated to be 16.5 nm/min. The binding energies of the XPS peaks were calibrated taking that of C1s peak (284.8 eV) as a standard reference. The O1s peaks were fitting by the XPSPEAK41 software. A Shirley typed background was used for the fitting. A Gaussian–Lorenz function was used to construct the component peaks for fitting the O1s peak. For quantitative analysis, the elemental sensitivity factors was set according to the reference for obtaining the atomic ratio in the film [22]. The transmittance (*T*) and reflectance (*R*) spectra in the wavelength of 400–1000 nm of the In_2_O_3_/glass specimens were measured using a spectrometer (MFS-630, Hong-Ming Technology, New Taipei City, Taiwan). The light was incident from the air background to the film during the measurement. The electrical properties of the In_2_O_3_ film were measured by a Hall effect measurement system (HMS5000, Side Semiconductor Technology, Shanghai, China). The measurement was carried out at room temperature. The strength of the magnetic field for the measurement is 0.556 T. The electrodes for the measurement were made of indium balls and arranged according to the van der Pauw configuration.

## 3. Results

**Figure 2:**
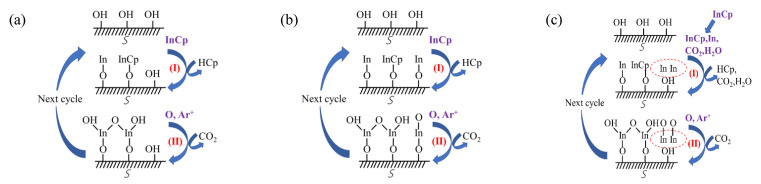
Deposition mechanisms of PEALD-In_2_O_3_ thin film at different substrate temperatures: (**a**) the thermal activation of the adsorbed precursors at low temperature; (**b**) self-limiting reaction with saturated adsorbed precursors at intermediate temperature; and (**c**) partially thermal decomposition of the precursors at high temperature.

Figure 2 shows a schematic description of the deposition mechanisms of the PEALD-In_2_O_3_ thin-film deposited at different substrate temperatures. The reaction can be described by the following formulas [13]:

S-OH + InCp → S-O-In-Cp_(1 − *x*)_ + xCpH↑ + (1 − *x*)/2H_2_↑ (3)

S-O-InCp_(1 − *x*)_ + O* → S-O-In-O(OH) + CO_2_ (4)

where S- represents the substrate surface. The symbol ↑ denotes the volatile gaseous phase. The chemical formula for InCp is In(CH)_5_. In the first half-reaction, the InCp with a ratio of *x* chemically reacts with surface hydroxyl (OH) groups to form O–In bonds and release x volatile cyclopentadiene (CpH) and (1 − *x*)/2 H_2_, leaving (1 − *x*) InCp that is physically adsorbed on the surface by van der Waals interaction force and does not participate in forming O–In bonds as described in formula (3). In the second half-reaction, oxygen radicals supplied by the O_2_ plasma react with the resulting surface to form In–O bonds, to regenerate initial hydroxyl ligands and release CO_2_ gas as described in formula (4). The film growth in different substrate temperature ranges is dominated by different mechanisms as depicted in Figure 2, which also lead to different behaviors in the film growth rate. Detailed description on the mechanisms would be discussed along with the growth rate mentioned below.

**Figure 3:**
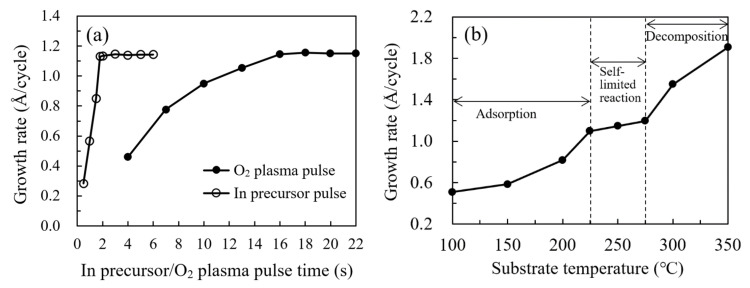
(**a**) Change in the growth rate of In_2_O_3_ films with the In precursor and O_2_ plasma pulse time; and (**b**) the growth rate of In_2_O_3_ films on a silicon wafer as a function of substrate temperature.

The saturated surface reaction between InCp and O2 plasma was confirmed at a substrate temperature of 250 oC, as depicted in Figure 3a. By increasing the InCp pulse time from 0.5 to 6 s, a saturated growth rate of about 1.15 Å/cycle could be achieved at In precursor pulse lengths over 1.8 s. A comparable growth rate about 1.15 Å/cycle was also found by varying the O2 plasma pulse time within 16−22 s at a fixed. In precursor pulse length of 2 s. According to these self-limiting reaction results, the optimal pulse conditions for the PEALD In2O3 process were determined to be a 2 s In precursor pulse, and a 16 s O2 plasma pulse. Figure 3b shows the growth rate of In_2_O_3_ films on a silicon wafer as a function of substrate temperature, where the growth rate was defined as the quotient of the film thickness to the cycle number. There are three distinct regimes of the curve with respect to the substrate temperature. Firstly, the growth rate significantly increases with substrate temperature at 100–200 °C as shown in Figure 2a. In this regime, the film growth is determined by the thermal activation of the adsorbed precursors, which transforms the physically adsorbed precursors to be chemically adsorbed. The chemically adsorbed precursors participated in forming O–In bonds and contributed to the film growth, whereas some precursors were physically adsorbed on the substrate surface by van der Waals interaction force and did not participate in forming O–In bonds. The ratio of chemically adsorbed precursors (*x*) increases whereas the ratio of physically adsorbed precursors (1−*x*) decreases with increasing substrate temperature. The increase in substrate temperature also enhances the reaction of the chemically adsorbed precursors with the oxygen radicals. Secondly, the growth rate seems to be saturated with an almost constant growth rate of around 1.15 Å/cycle was observed at 225–275 °C, which should be ascribed to the complete activation of the adsorbed InCp precursor with a ratio of x approaching 1. According to the saturated reaction results in Figure 3a, the film grows in a saturated self-limited reaction manner in this regime. Thirdly, at a high temperature of 300–350 °C as shown in Figure 2c, the film growth rate increases abruptly as the substrate temperature increases, which should be ascribed to the thermal decomposition of the InCp precursor. In this regime, a portion of the indium precursor decomposed and directly deposited on the substrate in the first-half reaction, then oxidized by the oxygen radicals in the second-half reaction, resulting in a CVD-like film growth mode with higher film growth rate. This variation trend of the growth rate with respect to substrate temperature is in accordance with the results reported by several groups [2,3]. The different dependence of the growth rate with respect to the substrate temperature corresponds to the different mechanisms depicted in Figure 2, which were also supported by various experimental results as mentioned afterward. The thicknesses of the In_2_O_3_ films grown at 100, 150, 200, 225, 250, 275, 300 and 350 °C are 20.4, 23.5, 32.7, 43.9, 45.8, 47.8, 61.9 and 76.2 nm, respectively, when executing 400 cycles in total. Representative specimens belong to different regimes were selected for further characterization to clarify the relation between the mechanisms and the film properties.

**Figure 4:**
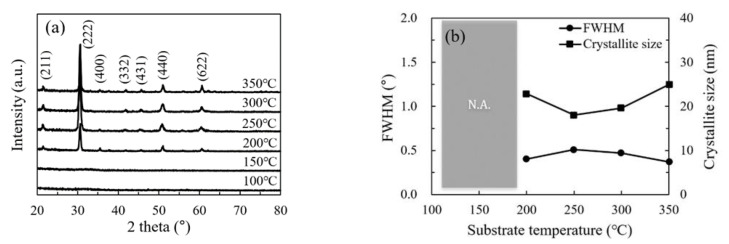
(**a**) The grazing incident X-ray diffraction (XRD) patterns of In_2_O_3_ films deposited at different substrate temperatures; (**b**) variation of full width at half maximum (FWHM) for the (2 2 2) peak and the size of the (2 2 2)-orientated crystallites with substrate temperature. N.A. is the abbreviation for not available.

The structural and morphological characteristics of the In_2_O_3_ films were investigated by GIXRD, FESEM and AFM. Figure 4a shows the GIXRD patterns of In_2_O_3_ films on silicon wafer. No diffraction peaks were observed for the In_2_O_3_ films deposited at low temperature (100 and 150 °C), indicating the films are amorphous. At such a low temperature, the precursors do not have adequate energy to migrate to the energetically favorable sites, leading to the amorphous phase. In addition, the large amount of physically adsorbed precursors may also play a role in the formation of amorphous phase as they may act as the steric hindrance that hinder the migration of the chemically adsorbed precursors. As the substrate temperature increases, the precursors gain more energy to migrate to the energetically favorable sites. Meanwhile, the steric hindrance introduced by the physically adsorbed precursors reduced with increasing substrate temperature. As a result, the In_2_O_3_ film transformed from amorphous to poly-crystalline at a temperature between 150 and 200 °C. Significant diffraction peaks were observed for the In_2_O_3_ films deposited at 200 °C and above. A similar amorphous-to-crystalline phase transition has been reported by several other groups [14,23]. These observed peaks origin from the diffraction by the (2 1 1), (2 2 2), (4 0 0), (3 3 2), (4 3 1), (4 4 0) and (6 2 2) planes of the In_2_O_3_ lattice with cubic bixbyite structure [14,16,17]. The strongest peak located at 2*θ* = 30.585° corresponds to the closest packing plane in the In_2_O_3_ lattice, implying that the deposited films have a compact structure. Figure 4b shows the FWHM value and average crystallite size with respect to the substrate temperature. The crystallite sizes for the films deposited at 100 °C and 150 °C are not available due to the amorphous nature of the films. The crystallite sizes for the films deposited at 200–350 °C show a weak dependence on the substrate temperature and varies in a small range of 19–25 nm.

**Figure 5:**
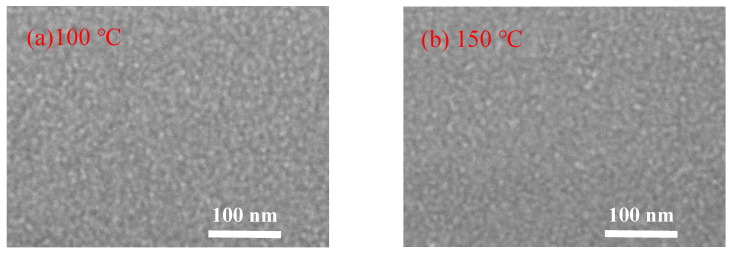

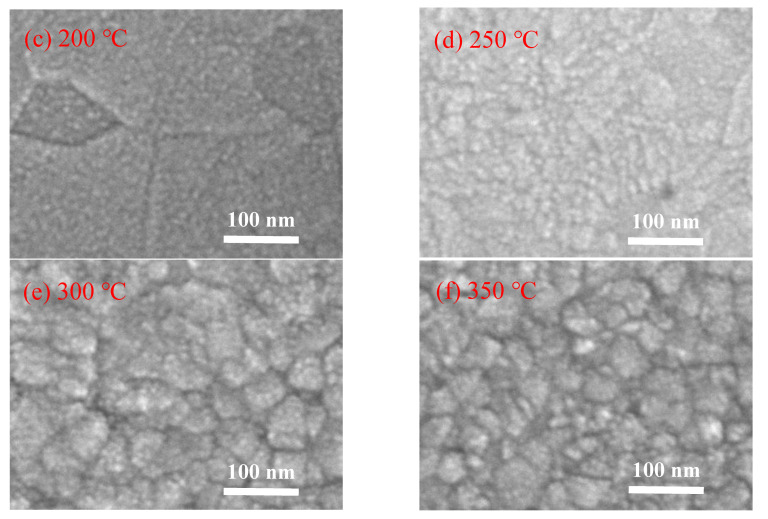
Field-emission scanning electron microscope (FESEM) images of PEALD-In_2_O_3_ films deposited at a substrate temperature of (**a**) 100 °C; (**b**) 150 °C; (**c**) 200 °C; (**d**) 250 °C; (**e**) 300 °C; and (**f**) 350 °C.

Figure 5 shows the FESEM images of the In_2_O_3_ films grown on silicon wafer. The In_2_O_3_ films deposited at low temperature (100 and 150 °C) exhibit a typical amorphous morphology. When deposited at 200 °C, the film seems to consist of plausible crystalline domains of tens nanometers, within which nanoscaled grains with unconspicuous grain boundaries were observed [23]. As the deposition temperature increases to 250 °C, the film exhibits a smooth surface with unconspicuous grain boundaries. As the substrate temperature further increases to a higher value of 300 °C and 350 °C, large grains with obvious grain boundaries were observed. The grain sizes observed from the FESEM images of the films deposited at high temperature are about 50 nm, which are significantly larger than the crystallite sizes deduced from the XRD results. Therefore, the larger grains may be clusters consisting of smaller crystallites. These results are in good agreement with the assumption of the thermal decomposition of the indium precursor and the CVD-like deposition mode at such a high substrate temperature, which leads to the cluster-like surface morphology.

**Figure 6:**
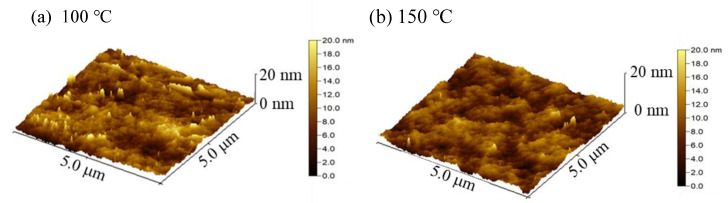

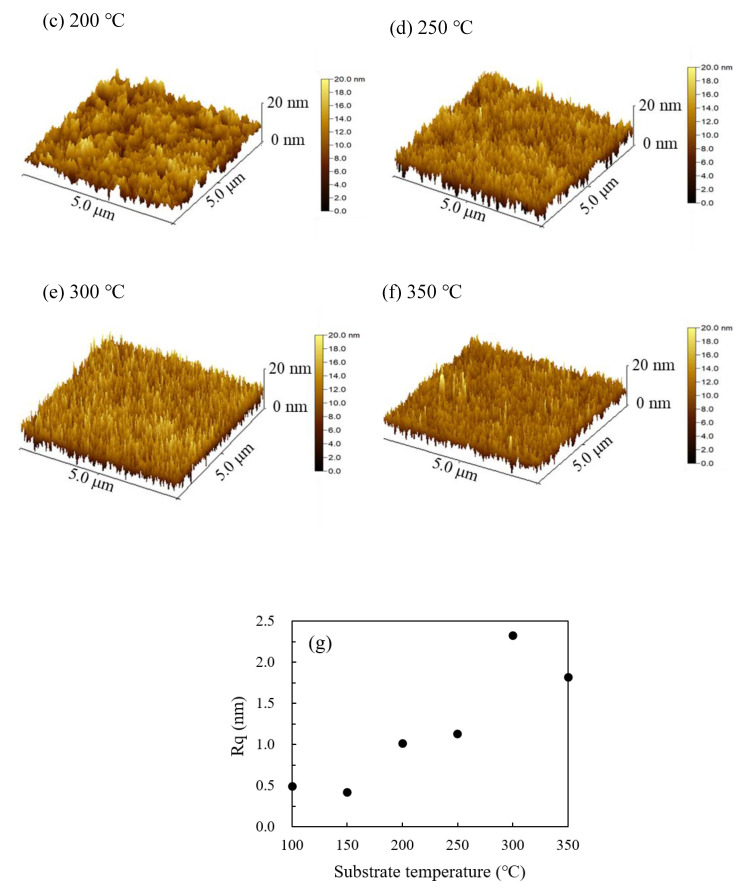
Three-dimension atomic force microscopy (AFM) images of PEALD-In_2_O_3_ films deposited at a substrate temperature of (**a**) 100 °C; (**b**) 150 °C; (**c**) 200 °C; (**d**) 250 °C; (**e**) 300 °C; and (**f**) 350 °C; (**g**) the variation of root-mean-square roughness (*R*_q_) of the PEALD-In_2_O_3_ films with substrate temperature.

Figure 6a–f shows the three-dimension AFM images of the In_2_O_3_ films deposited at various substrate temperatures. The root-mean-square roughness (*R*_q_) was also extracted from the images and plotted in Figure 6g. The In_2_O_3_ films deposited at low temperature (100 °C and 150 °C) exhibit smooth surface with an *R*_q_ value of around 0.5 nm owing to the uniform amorphous phase. The film surfaces become rougher with an *R*_q_ value of around 1–1.2 nm when deposited at intermediate temperature (200 °C and 250 °C) due to the crystallization. The roughness of the film surface increases significantly when deposited at a high temperature (300 °C and 350 °C) due to the existence of the coarse clusters. Overall, the development of structural and morphological characteristics agrees well with the mechanisms of the film growth at different substrate temperature ranges.

**Figure 7:**
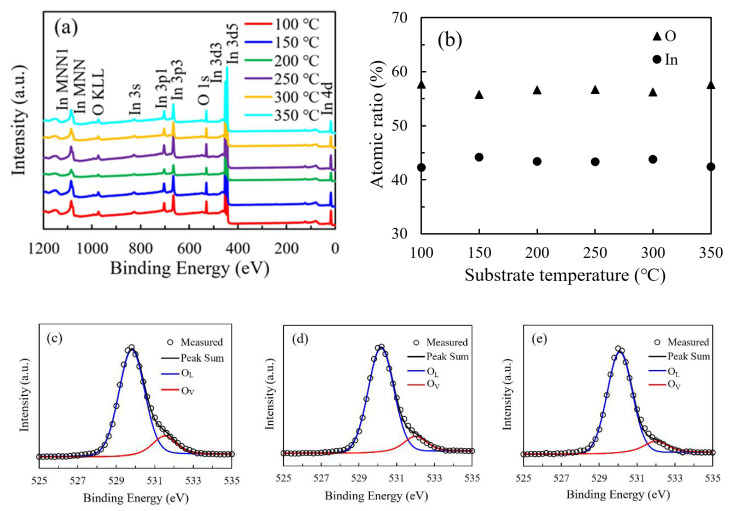

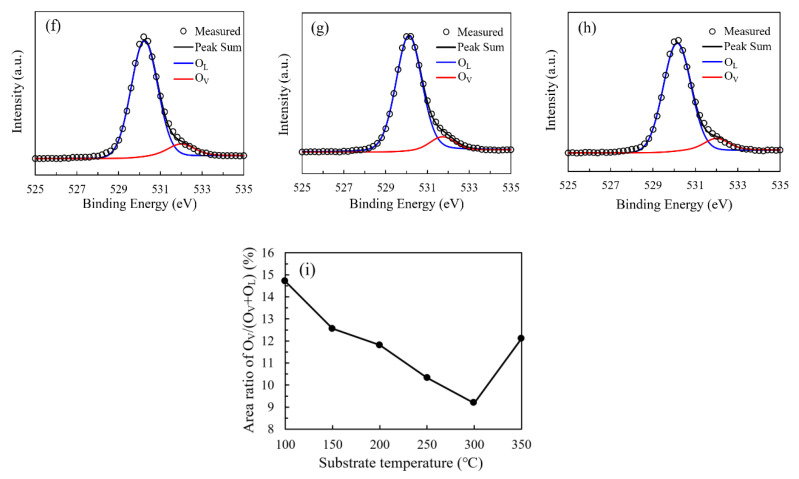
(**a**) The broadly surveyed XPS spectra of the In_2_O_3_ films; (**b**) the variation in the relative atomic ratio of In and O elements with substrate temperature; (**c**–**h**) the high-resolution spectra of O 1s peak of the PEALD-In_2_O_3_ films deposited at 100–350 °C; (**i**) the variation of area ratio of O_V_/(O_V_+O_L_) with substrate temperature.

Figure 7a shows the broadly surveyed XPS spectra of the In_2_O_3_ films. The observed peaks correspond to the In or O element. The variation of the relative atomic ratio of In and O elements with substrate temperature was shown in Figure 7b. All the In_2_O_3_ films were In-rich/O-poor with the In-atom-ratio higher than 40% an O-atom-ratio lower than 60% when compared with the stoichiometric In_2_O_3_. The O atomic ratio is almost constant with values of 55.8%–56.7% in the temperature range of 150–300 °C. Otherwise, the O atomic ratio is slightly higher with values of 57.7% and 57.6% for the In_2_O_3_ films deposited at 100 °C and 350 °C, possibly due to the great deviation from the saturated ALD growth mode at these two extreme substrate temperatures. The In atomic ratio exhibit opposite variation tendency. The higher O content for the films deposited at 100 °C and 350 °C should be ascribed to the relative shortage of the chemically absorbed precursors due to the low energy at low substrate temperature and the thermal decomposition of the precursor at high substrate temperature. In contrast, the supply of O_2_ plasma oxidant is relatively adequate, resulting in the higher O content for these films. The O 1s peak was deconvoluted into two components located at around 529.8 eV (O_L_) and 531.4 eV (O_V_), respectively, related to the O atoms in a stoichiometric and oxygen-deficient In_2_O_3_ lattice as shown in Figure 7c–h [11,14]. The area ratio of O_V_/(O_V_+O_L_) was used to reflect the amount of oxygen vacancies and shown in Figure 7i. The area ratio continuously decreases from 14.7% to 9.1% as the substrate temperature increases from 100 °C to 300 °C, suggesting that the ratio of chemically adsorbed precursors increases, and hence more oxygen atoms participate in forming In–O bonds as the reaction energy provided by heating the substrate increases, resulting in a more perfect In_2_O_3_ lattice. However, the total O-content in the film exhibits a weak dependence on the substrate temperature. There is a seeming paradox between the variation trends of the oxygen vacancy concentration and the total O-content. In fact, they are unnecessarily related to each other since the oxygen atoms may exist as lattice oxygen, oxygen interstitials (O_i_) or OH ligands with binding energies located at 529.8, 532.4 and 531.9 eV, respectively [24,25]. Although the (O_i_) or OH components cannot be distinguished from the O1s peak, there are still possibilities that more oxygen atoms may exist as oxygen interstitials or OH ligands in the film when deposited at a relatively low substrate temperature due to the low ratio of chemically adsorbed precursors. As a result, a poorer In_2_O_3_ lattice was formed. As the substrate temperature increases, the oxygen interstitials and OH ligands reduce, meanwhile more oxygen atoms incorporate in forming the In_2_O_3_ lattice. Therefore, it is reasonable for the total O-content to keep at an almost constant level. As the substrate temperature further increases to 350 °C, the area ratio increases to 12.1%, possibly due to the severe decomposition of the precursors at such a high temperature.

**Figure 8:**
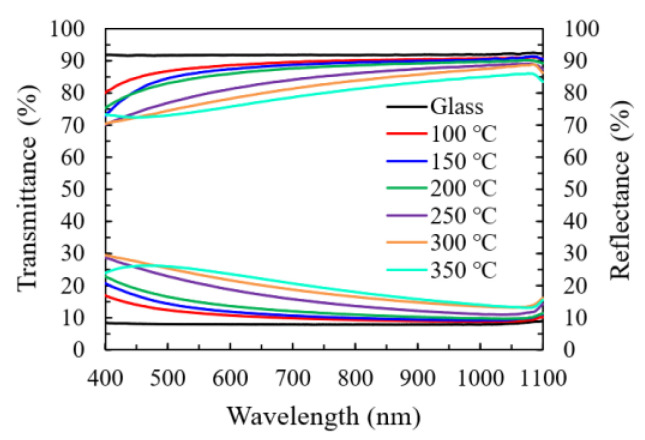
The transmittance and reflectance spectra of the In_2_O_3_/glass specimens prepared with different substrate temperatures.

Figure 8a shows the transmittance and reflectance spectra of In_2_O_3_ films grown on the glass substrate at various temperatures. Overall, the specimens all exhibit a high transmittance (70%–90%) in the visible and near-infrared light range (400–1000 nm). The variation of transmittance spectra should be primarily ascribed to the change in the reflection conditions since they exhibit a reverse relation to the reflectance spectra. The factors that affect the reflection conditions include a change in film thickness and refractive index in this study.

**Figure 9:**
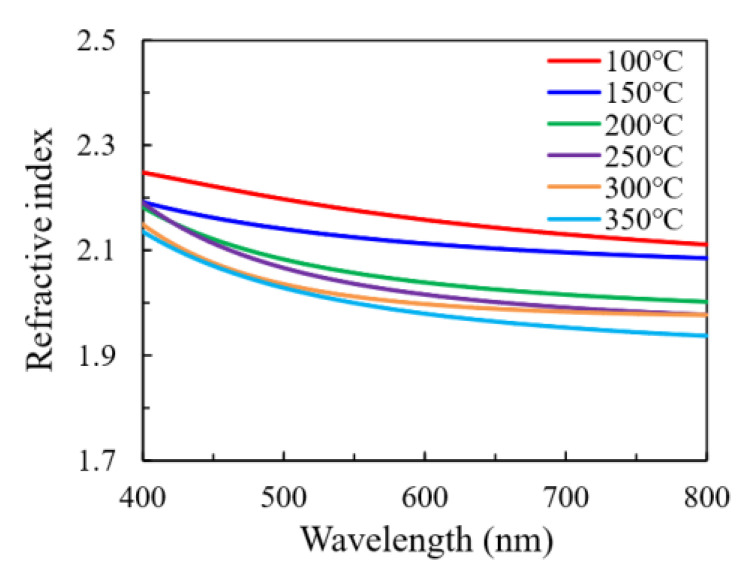
The wavelength-dependent refractive index of In_2_O_3_ films deposited at different substrate temperatures.

Figure 9 shows the wavelength-dependent refractive index (*n*) of In_2_O_3_ films. Generally, the refractive index of the thin film can be affected by the crystallinity, constituent and density of the film [26,27,28]. In this case, the crystalline fraction seems to have primary influence on the refractive index. In addition, the variation in the constituent, the chemical states of constituent elements and the density of the film may play a role in influencing the refractive index for the films with the same crystal phase. Broadly speaking, the In_2_O_3_ films deposited at low temperature (100–150 °C) are amorphous and exhibit a relatively high refractive index. The observation from the SEM results also suggests a compact structure of these films, which rationalizes their high refractive indices. The minor deviation between the refractive indices of the films deposited at 100 °C and 150 °C is possibly related to the variation in the film constituent and the chemical states of the O elements. As observed by the XPS, the film deposited at 100 °C has higher O-content and more O atoms may exist as oxygen interstitials or OH ligands. In addition, the possible-existing carbon content that has been reported to decrease with increasing substrate temperature may have influence on the refractive index [14,18]. A sharp decrease in refractive index at 150–200 °C was observed, which should be ascribed to the amorphous-to-crystalline transition. The refractive indices for the films deposited at 200 °C and 250 °C are very close to each other as the substrate temperature located in the ALD window with self-limiting reaction. However, the refractive index further decreases as the substrate temperature increases to 300 °C and 350 °C, which should be ascribed to the decomposition of the indium precursors. As has been observed by the FESEM results, large clusters with significant grain boundaries have been observed, which leads to a coarse film with a loose structure.

**Figure 10:**
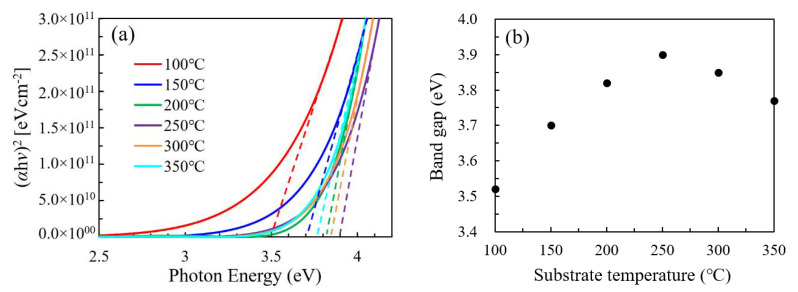
(**a**) Tauc plots and (**b**) optical band gap for the In_2_O_3_ films deposited at different substrate temperatures.

The Tauc plots and optical band gap of the In_2_O_3_ films was plotted in Figure 10a,b, respectively. The optical band gap significantly increases as the substrate temperature increases from 100 to 250 °C and reaches the highest value at 250 °C. Then, it slightly decreases as the substrate temperature further increases to 300–350 °C. The narrow optical band gap for the In_2_O_3_ films deposited at a low temperature (100 °C and 150 °C) should be due to the highly disordered amorphous structure of the film, which may induce a large amount of defect states near the conduction band tail. The significant increase in the optical band gap with substrate temperature at 100–200 °C should be ascribed to the amorphous-to-crystalline transition as indicated by the XRD results, which greatly reduce the defect states near the conduction band tail. The slight decrease in optical band gap at 300–350 °C should be ascribed to the decomposition of the precursors, which may induce more defect states near the conduction band tail. The variation of defect states is broadly in line with the variation of oxygen vacancy defects as indicated by the area ratio of OV/(OV+OL) shown in Figure 6i.

**Figure 11:**
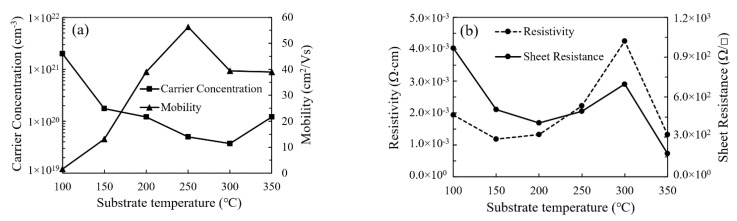
The variation of (**a**) the carrier concentration, mobility and (**b**) resistivity of In_2_O_3_ films with substrate temperature.

Figure 11a shows the variation of carrier concentration and mobility of In_2_O_3_ film with substrate temperature. The variation of carrier concentration exhibits similar trends to that of the amount of oxygen vacancies. This is accordant to the common viewpoint that oxygen vacancies act as one of the major origins for the n-type conductivity in In_2_O_3_ film [29,30,31]. The In_2_O_3_ film deposited at 100 °C has a very low carrier mobility of 1.6 cm^2^/V·s due to the existence of a large amount of defects. However, the carrier mobility significantly increases with increasing substrate temperature and reaches the highest value of 56.3 cm^2^/V·s when deposited at 250 °C owing to the reduction in defects and the formation of more perfect In_2_O_3_ lattice. Nevertheless, it decreased to around 40 cm^2^/V·s when the substrate temperature further increased to 300–350 °C, possibly due to the scattering by the obvious grain boundaries as observed by the SEM. The variation of film resistivity (*ρ*) and sheet resistance (*R*_s_) with substrate temperature is shown in Figure 11b. The relation between film resistivity and sheet resistance can be expressed as: *ρ* = *R*_s_·*t*. The sheet resistance is film-thickness-dependent while the film resistivity is much less influenced by the film thickness. Thus, we focus on the film resistivity to reflect the electrical properties of the films. The resistivity of the In_2_O_3_ film is determined by the carrier concentration and mobility. Overall, the film resistivity increases with increasing substrate temperature and reaches the highest value of 4.25 × 10^−3^ Ω·cm at 300 °C due to the decrease in carrier concentration. The relatively high resistivity of the film deposited at 100 °C is due to the low carrier mobility. Then, it reduces to 1.33 × 10^−3^ Ω·cm at 350 °C due to the increase in carrier concentration.

## 4. Conclusions

In this paper, In_2_O_3_ film was prepared at different substrate temperature by an ALD process using O_2_ plasma as an oxidant. When deposited at a low temperature of 100–150 °C, the films were amorphous. A high refractive index, a low optical band gap, a high carrier concentration and low carrier mobility were observed for these films. The films transformed to crystalline at 150–200 °C. When deposited at an intermediate temperature of 225–275 °C, an ALD window was observed and a reasonable film growth rate of around 1.15 Å/cycle was achieved. The resulting films exhibit a comparable refractive index of around 2.0–2.1 at 630 nm, a comparable optical band gap of 3.52–3.88 eV, a relatively low carrier concentration of 5.0 × 10^19^ cm^3^ and the highest carrier mobility of 56.3 cm^2^/V·s when deposited in the ALD window. When deposited at a high temperature of 300–350 °C, a portion of the precursors decomposed, hence the CVD-like deposition of the film was observed, resulting in a rough film with coarse grains, obvious grain boundaries and a reduced carrier mobility was detected. The growth mechanism and properties of the In_2_O_3_ films prepared by plasma-enhanced ALD can be efficiently tuned by substrate temperature.

## Figures and Tables

**Table 1:** Deposition parameters of the plasma-enhanced ALD (PEALD) In_2_O_3_ films.

Parameter	Value
Bubbler temperature (°C)	90
Substrate temperature (°C)	100–350
InCp pulse time (s)	2
InCp purge time (s)	6
InCp carry gas flow rate (sccm)	120
InCp dilute gas flow rate (sccm)	400
O_2_ pulse time (s)	16
O_2_ purge time (s)	5
Ar flow rate (sccm)	80
O_2_ flow rate (sccm)	380
O_2_ plasma power (W)	3000
